# The Columbia Hospital and Lying-in Asylum

**Published:** 1877-11-20

**Authors:** 


The Columbia Hospital and Lying-In Asylum. A Government Institution. Its past and present management. By a citizen of Washington, D. C.

